# Predictors of Tuberculous Meningitis Mortality Among Persons with HIV in Mozambique

**DOI:** 10.3390/tropicalmed10100276

**Published:** 2025-09-24

**Authors:** Edy Nacarapa, Isabelle Munyangaju, Dulce Osório, Jose-Manuel Ramos-Rincon

**Affiliations:** 1 Carmelo “TB/HIV Reference” Hospital of Chokwe—The Daughters of Charity, Saint Vincent de Paul, Chokwe 1204, Mozambique; 2 Tinpswalo Research Association to Fight AIDS and TB, Chokwe P.O. Box 35, Mozambique; imunyangaju@gmail.com (I.M.); dulceosorio92@gmail.com (D.O.); 3 Internal Medicine Department, Alicante General University Hospital and University Miguel Hernandez de Elche (UMH), 03202 Elche, Spain; jose.ramosr@umh.es

**Keywords:** tuberculous meningitis, HIV, mortality, malnutrition, Mozambique

## Abstract

*Background*: Tuberculous meningitis (TBM) is the most severe form of tuberculosis and is associated with high morbidity and mortality, especially in resource-limited settings. In Mozambique, where both tuberculosis and HIV are highly prevalent, TBM poses significant diagnostic and therapeutic challenges. This study aimed to describe the clinical characteristics and to identify predictors of TBM mortality among persons living with HIV (PLWH) in a rural hospital in Mozambique. *Methods*: We conducted a retrospective cohort study at Carmelo Hospital of Chokwe (CHC) between 2015 and 2020. We included 372 PLWH diagnosed with TBM (PTBM); data on demographics, clinical presentation, and laboratory findings were extracted from patient records. TBM diagnosis was considered for confirmed cases based on a hospital-adapted algorithm incorporating clinical features, cerebrospinal fluid (CSF) analysis, TB-LAM, and Xpert MTB/RIF testing. Cox proportional hazard models were used to identify independent predictors of mortality, and Kaplan–Meier survival curves with log-rank tests were used to assess survival differences across clinical subgroups. Significance was considered at a *p* value ≤ 0.05 with an adjusted hazard ratio (AHR) 95% CI in the multivariate analysis. *Results*: Overall, 372 PTBM contributed to a total of 3720 person-months (PM) of treatment follow-up, corresponding to a mortality incidence of 3.76 deaths per 100 person-months. Factors independently associated with increased mortality included male sex (adjusted hazard ratio [aHR]: 1.80; 95% CI: 1.21–2.68; *p* = 0.004), BMI < 18.5 kg/m^2^ (aHR: 2.84; 95% CI: 1.46–5.55; *p* = 0.002), Immunovirological failure to ART (aHR: 2.86; 95% CI: 1.56–5.23; *p* = 0.001), CSF opening pressure >40 cmH_2_O (aHR: 2.67; 95% CI: 1.46–4.86; *p* = 0.001), and TBM severity grading III (aHR: 4.59; 95% CI: 1.79–11.76; *p* = 0.001). TBM involving other organs also significantly worsened survival (aHR: 2.03; 95% CI: 1.27–3.25; *p* = 0.003). *Conclusions*: TBM mortality in PLWH was driven by ART failure, high CSF pressure, and malnutrition. Male sex and severe neurology also increased risk. Urgent interventions are proposed: optimize ART, manage intracranial pressure, provide nutritional support, and use corticosteroids. An integrated care approach is essential to improving survival in resource-limited settings.

## 1. Introduction

Tuberculous meningitis (TBM) is the most devastating manifestation of extrapulmonary tuberculosis, accounting for a significant proportion of tuberculosis-related deaths and long-term neurological disability [1,2]. The disease arises when *Mycobacterium tuberculosis* invades the central nervous system, triggering a granulomatous inflammatory response in the meninges [3,4,5]. This process can result in hydrocephalus, vasculitis, infarctions, and raised intracranial pressure, contributing to its high case fatality and the potential for profound neurocognitive sequelae [3,6]. Early diagnosis and initiation of anti-tuberculosis therapy, often supplemented with corticosteroids, are essential to improving outcomes, yet diagnostic delays are common due to the nonspecific early symptoms and limited diagnostic capacity in many healthcare settings [1,7].

Globally, TBM poses a major challenge in tuberculosis-endemic regions, particularly in sub-Saharan Africa (sSA) where the burden of HIV co-infection exacerbates disease severity and complicates clinical management [6,8]. TB and HIV are deadly syndemic in humans; HIV significantly increases the risk of TB reactivation by 20–30 times, contributes to 25% of global HIV-related deaths with a doubled fatality rate in co-infected individuals (37% vs. 16%), and disproportionately affects sSA, which accounts for 79% of global HIV-TB cases [9].

Persons living with HIV (PLWH) are at higher risk of developing disseminated TB, including TBM, and they have a poorer prognosis due to immunosuppression and the increased likelihood of drug-resistant strains [10,11]. In resource-limited environments, the lack of access to cerebrospinal fluid (CSF) culture, imaging, and rapid molecular diagnostics such as Xpert MTB/RIF Ultra compounds the difficulty of confirming TBM, leading to reliance on clinical criteria and empiric treatment [3,10]. Despite these challenges, studies focusing specifically on TBM in rural African settings remain limited, especially regarding mortality outcomes and predictor profiles.

Mozambique is among the 30 countries with the highest tuberculosis rates (incidence of 361 per 100,000 population) and TB/HIV burden (incidence of 83 per 100,000 population), with significant disparities in healthcare infrastructure between urban and rural areas [8,12]. In rural hospitals, the diagnosis and management of TBM are often hindered by insufficient laboratory support, shortages of trained personnel, and delays in referral pathways. While national TB control programs have made progress in expanding diagnostic and treatment services [13,14], TBM remains under-recognized and under-reported. The absence of local epidemiological data on TBM contributes to the lack of targeted clinical guidelines for rural health facilities and hampers efforts to improve case detection and survival.

This study aimed to describe the clinical characteristics and to identify predictors of TBM mortality among PLWH in a rural hospital of Mozambique. By providing evidence from a real-world rural setting, the study seeks to contribute to the limited body of knowledge on TBM in high-burden, resource-constrained environments.

## 2. Materials and Methods

### 2.1. Study Design and Setting

This was a retrospective cohort study conducted at Carmelo Hospital of Chókwè (CHC), a rural referral facility in southern Mozambique. The hospital serves a predominantly rural population with a high burden of TB and HIV. The study period spanned six years, from January 2015 to December 2020, and focused specifically on cases of TBM diagnosed and managed at the facility.

### 2.2. Study Population and Case Selection

**Figure 1:**
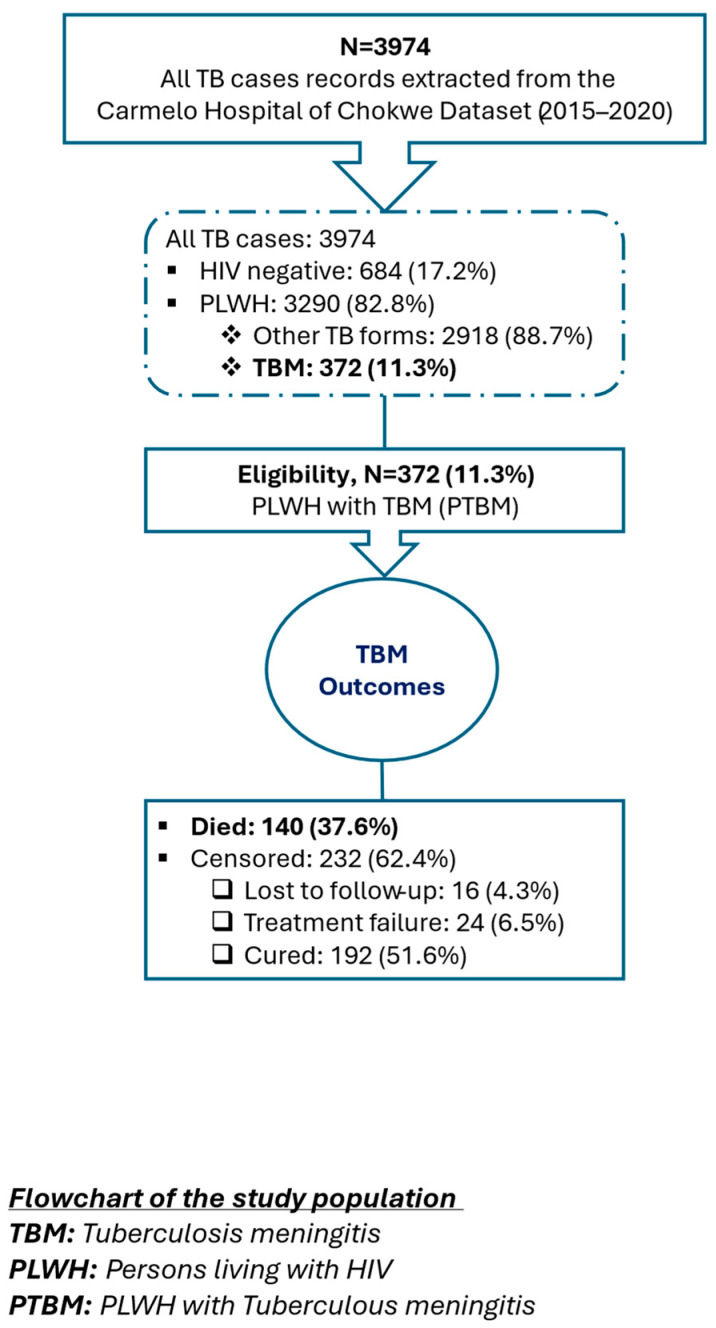
Flowchart of study population.

**Figure 2:**
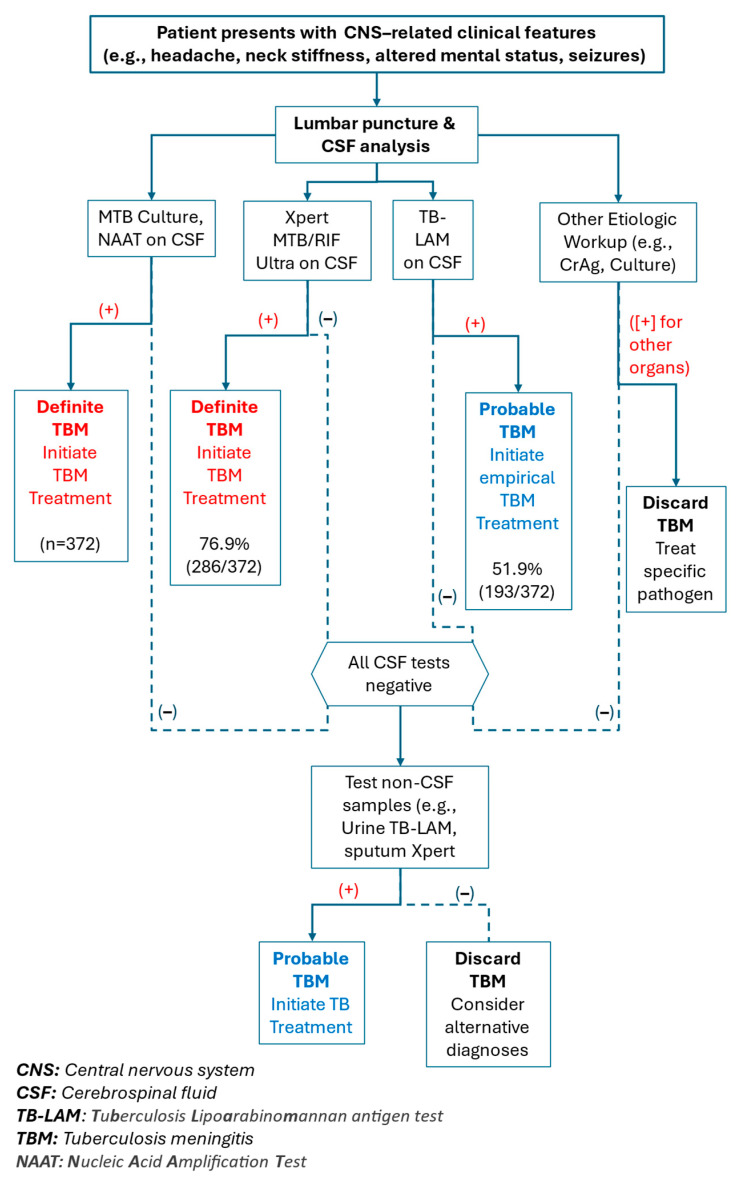
Screening algorithm for tuberculous meningitis adjusted to Carmelo Hospital in Chokwe.

Medical records of all TB patients, including adults and children registered in the hospital’s tuberculosis database during the study period, between 2015 and 2020, were reviewed. A total of 3974 TB cases were recorded. From these, 3290/3974 (82.8%) were TB/HIV coinfected, from which 372/3290 (11.3%) PLWH with a confirmed diagnosis of TBM (PTBM) were eligible for the analysis. Cases of pulmonary TB, TBM with HIV negative status and other forms of TB were excluded 3602/3974 (90.6%) (Figure 1). The sample consisted of all eligible PLWH registered as having TBM; therefore, no formal sampling calculations were applied. TBM cases were identified based on clinical documentation in the medical records following a hospital-adapted screening and diagnostic algorithm (Figure 2).

#### 2.2.1. TB Management at CHC

In clinical practice at CHC, physicians routinely initiated the empirical anti-tuberculosis treatment while awaiting laboratory confirmation. This standard approach explains why the other types of tuberculosis are mentioned in Figure 1 (pulmonary, extrapulmonary TB, single- or multiple-organ involvement), considering probable and possible tuberculosis diagnoses. This reflects the real-life diagnostic challenges where definitive confirmation is often delayed, but early treatment is crucial. Following standard protocols, all TB-diagnosed patients received WHO-recommended anti-TB regimens, irrespective of their potential inclusion in the study population.

The first-line treatment for TBM consisted of a 2-month intensive phase with rifampicin, isoniazid, pyrazinamide, and ethambutol, followed by a 7–10-month continuation phase with rifampicin and isoniazid, alongside adjunctive dexamethasone, while HIV-coinfected patients-initiated ART within 2–8 weeks of TB treatment [15].

For the specific purposes of our analysis, we selectively extracted only those cases with microbiologically confirmed tuberculous meningitis. This stringent criterion ensured diagnostic certainty in our study cohort, although it represented a subset of the broader TB cases that were managed clinically. The decision to focus on definitively diagnosed TBM cases allowed for more precise outcome assessments while acknowledging that, in actual practice, many patients are treated empirically based on clinical suspicion alone.

#### 2.2.2. TBM Screening and Diagnostic Criteria

At CHC, the diagnosis of TBM followed a structured screening algorithm integrating clinical assessment with stepwise laboratory testing. Patients presenting with central nervous system (CNS)-related clinical features—such as headache, neck stiffness, altered mental status, or seizures—underwent lumbar puncture (LP) for CSF testing.

Initial CSF testing included lateral flow lipoarabinomannan (TB-LAM), Xpert MTB/RIF Ultra, Culture and/or NAAT (Nucleic Acid Amplification Test). A positive Xpert MTB/RIF Ultra, Culture and/or NAAT result was considered confirmatory and led to a classification of definite TBM and immediate treatment initiation. A positive CSF TB-LAM result, while supportive, was considered indicative of probable TBM due to its lower sensitivity in CSF and initiated empirical treatment pending further investigation.

If both initial tests were negative, further diagnostic steps were pursued. These included CSF analysis for other aetiologies such as cryptococcal antigen (CrAg) and culture. Positive findings for non-TB pathogens resulted in treatment specific to the identified condition, and TBM was ruled out.

In cases where all CSF tests (TB-LAM, Xpert, CrAg, culture, NAAT) were negative, additional testing using non-CSF samples was performed. Urine TB-LAM or sputum Xpert MTB/RIF Ultra tests were used to evaluate for other organ involvement in TB. A positive result in these tests prompted a classification of probable TBM and the initiation of TB treatment, while negative results led to the exclusion of TBM.

This diagnostic algorithm allowed for a pragmatic, tiered approach to TBM diagnosis in a resource-limited setting, supporting rapid treatment decisions while accounting for differential diagnoses (Figure 2).

For the specific purposes of this analysis, only laboratory-confirmed (definite) TBM cases, based on a positive CSF Xpert MTB/RIF Ultra, culture or NAAT, were included.

### 2.3. Data Collection and Variables

A standardized data extraction tool was used to collect information from paper-based medical records, including digitized electronic archives.

The primary outcome was mortality during TBM treatment follow-up. This outcome was categorized as either death or censored observation (encompassing treatment completion/cure, loss to follow-up, and treatment failure) [16]. Independent variables were categorized into six fields. First, the length of TBM treatment follow-up (start and end date). Second, demographic profile: biological sex (women and men), age (according to standard age group for TB reporting [17]). Third, nutritional status: body mass index (BMI). Fourth, HIV immunovirological status to ART (ART history and virological suppression): optimal immunovirological response (OIVR), immunological non-responders (INR), immunovirological failure (IVF). Fifth, anatomic sites: TBM only, TBM plus other organs. Sixth, clinical features: CSF opening pressure [OP]; meningeal signs (neck stiffness, severe headache, photophobia, vomiting); focal central nervous system (CNS) deficits (cranial nerve palsies [III, VI, VII], aphasia, hemiparesis); altered mental status (AMS (confusion, drowsiness)); seizures; TBM severity grading (per Modified British Medical Research Council (BMRC) scale [18]).

#### 2.3.1. Conceptual Definition of Immunovirological Status (IVS) to ART

According to several literature reviews [19,20,21] and in alignment with the WHO Guidelines on ART therapy [22], as well as approaches implemented in Mozambique [23,24,25], the adopted conceptual definition of IVS is as follows:

#### 2.3.2. Optimal Immunovirological Response (OIVR) to ART

PLHIV on ART at least ≥3 months with suppressed HIV viremia and acceptable immune reconstitution CD4+ cell count > 350 cells/mm^3^. Viral suppression is defined as achieving an HIV viral load (VL) level less than 200 copies per milliliter of blood (VL < 200 cop/mL) [26,27,28].

#### 2.3.3. Immunological Non-Responders (INR) to ART

PLHIV on ART for at least ≥2 years who are unable to normalize their CD4+ T cell count to >500 cells/µL count even with persistent virological suppression [29], generally defined as having a CD4+ cell count < 350 cells/µL despite suppressed HIV-1 RNA [20].

#### 2.3.4. Immunovirological Failure (IVF) to ART

PLHIV on ART at least ≥3 months with unsuppressed HIV viremia, regardless of CD4 count cell [22,25].

### 2.4. Outcomes Definitions

#### 2.4.1. Died TBM

Patients with TBM who died during hospitalization or TB treatment.

#### 2.4.2. Censored TBM

- *Cured TBM:*
  ▪ *Clinical cure:* PTBM can be declared cured after completing a full course of anti-TB therapy (typically 9–12 months) with clinical resolution of symptoms (no fever, improved neurological function).
  ▪ *Microbiological cure:* confirmation via negative CSF cultures or PCR (e.g., Xpert MTB/RIF) after 2–3 months of treatment.
- *Treatment failure:* Clinical deterioration or persistent symptoms despite TB treatment.
- *Lost to follow-up*: Patients who discontinued treatment or were transferred to other facilities with unknown outcome.

### 2.5. Statistical Analysis

Statistical analysis was performed using the Statistical Package for the Social Sciences (SPSS) version 25. The primary outcome was the incidence of mortality over the person-time accrued from the date of starting (study enrolment) to the end date.

First, to describe participants’ baseline characteristics, we calculated frequencies and proportions for categorical data and median and interquartile range (IQR) for normally distributed data. Then, we calculated the mortality rate using the number of patients who experienced death during study follow-up divided by the person-time (months) at risk throughout the observation period, prior to the outcome of the respective cohort. Next, we conducted a Kaplan–Meier analysis to evaluate the period during TBM treatment until time of death, as well as using the log-rank (Mantel–Cox) test to compare the differences between groups. Subsequently, we compared the proportion of patients who died according to exposure variables using crude and adjusted Cox regression modelling, reporting adjusted hazard ratios (aHR) with corresponding 95% confidence intervals (CIs). Variables with *p*-value less than 0.2 in the univariate analyses were entered into the multivariable model. The proportional hazards assumption was assessed using Schoenfeld residuals and log–log plots.

## 3. Results

### 3.1. Baseline Demographic and Clinical Profile

A total of 372 PLWH with laboratory-confirmed TBM were included in the study between 2015 and 2020. Among the 372 PTBM with laboratory-confirmed definite TBM, the diagnostic yield of the various tests was as follows: CSF Xpert MTB/RIF Ultra was positive in 286 (76.9%) cases, while CSF TB-LAM was positive in only 193 (51.9%) cases. All cases were confirmed by either a positive NAAT (Xpert) result or a positive mycobacterial culture (Figure 2).

Demographically, 56.5% (*n* = 210) of 372 were men, and 37.6% (140) were aged 35–44 years, of whom the median age was 36 (IQR 29; 42). Median TBM treatment follow-up was 10 months (IQR 2; 20).

Undernutrition was prevalent, with 72.8% (*n* = 271) of patients presenting with a BMI < 18.5 kg/m^2^. At the time of TBM diagnosis, 83.1% (*n* = 309) of the cohort was on antiretroviral therapy (ART), with varied immunological and virological responses, and the remaining 16.9% (*n* = 63) of patients started ART after the anti-TB treatment (ATT) follow-up period. Isolated TBM was present in 84.1% (*n* = 313) of PTBM, while 15.9% (*n* = 59) of them had TBM with additional organs involvement.

Neurological symptoms were common with meningeal signs such as neck stiffness, photophobia, and vomiting observed in 74.2% (*n* = 276) of PTBM; altered mental status in 32.3% (*n* = 120); and focal neurological deficits in 11.3% (*n* = 42). TBM severity grade III (BMRC Scale) was reported in 32.8% (*n* = 122) of PTBM. Elevated CSF opening pressure was frequently observed, with 27.9% (*n* = 102) of PTBM presenting with markedly elevated pressures (>40 cmH_2_O) and 34.3% (*n* = 128) with moderate elevation (21–39 cmH_2_O). (Table 1).

### 3.2. Predictors of Mortality in Tuberculous Meningitis

In this study, the 372 PTBM contributed a cumulative total of 3720 person-months of follow-up, 140 PTBM died, corresponding to an overall mortality rate of 37.6% and an incidence of 3.76 deaths per 100 person-months (95% CI: 3.14–3.76). A multivariable Cox proportional regression model identified several independent predictors of mortality (Table 2).

Male sex was significantly associated with increased risk of death. Men had a 1.80-fold higher hazard of mortality compared to women (aHR: 1.80; 95% CI: 1.21–2.68; *p* = 0.004). Undernutrition, defined as a BMI ≤ 18.5 kg/m^2^, was another strong predictor. Compared to PTBM with BMI > 18.6 kg/m^2^, those who were undernourished had a mortality risk nearly three times higher (aHR: 2.84; 95% CI: 1.46–5.55; *p* = 0.002).

ART-related immunological and virological status prior to TB treatment initiation were also significantly associated with outcomes. PTBM with an optimal immunological response had the lowest mortality risk. In contrast, those with virological failure to ART demonstrated nearly three times higher mortality risk (aHR: 2.86; 95% CI: 1.56–5.23; *p* = 0.001). Similarly, those who were immunological non-responders, as well as those who had initiated ART within 90 days of starting TB treatment, had a mortality risk nearly two times higher, with aHRs of 2.06 (95% CI: 1.23–3.46; *p* = 0.006) and 2.09 (95% CI: 1.24–3.52; *p* = 0.006), respectively.

CSF OP was a strong predictor of mortality. Compared to PTBM with CSF OP ≤ 20 cmH_2_O, those with CSF OP ≥ 40 cmH_2_O had the highest mortality risk, with aHR of 2.67 (95% CI: 1.46–4.86; *p* < 0.001).

Compared to PTBM without meningeal signs, those who presented meningeal signs had a nearly three times higher mortality risk (aHR: 2.66; 95% CI: 1.60–4.41; *p* < 0.001). In addition, PTBM with other organs involved were significantly associated with a mortality risk that was about two times higher compared to those with isolated TBM (aHR: 2.03; 95% CI: 1.27–3.25; *p* = 0.003).

Lastly, PTBM with TBM severity grade III (BMRC scale) had nearly five times the mortality risk (aHR: 4.59; 95% CI: 1.79–11.76; *p* = 0.001), compared to those with TBM grade I.

No significant associations were found between TBM mortality and baseline characteristics such as age group, focal neurological deficits, altered mental status (AMS), and seizures (Table 2).

### 3.3. Cumulative Mortality Rate (Time-to-Death for Non-Survivors): Temporal Mortality Patterns

**Figure 3:**
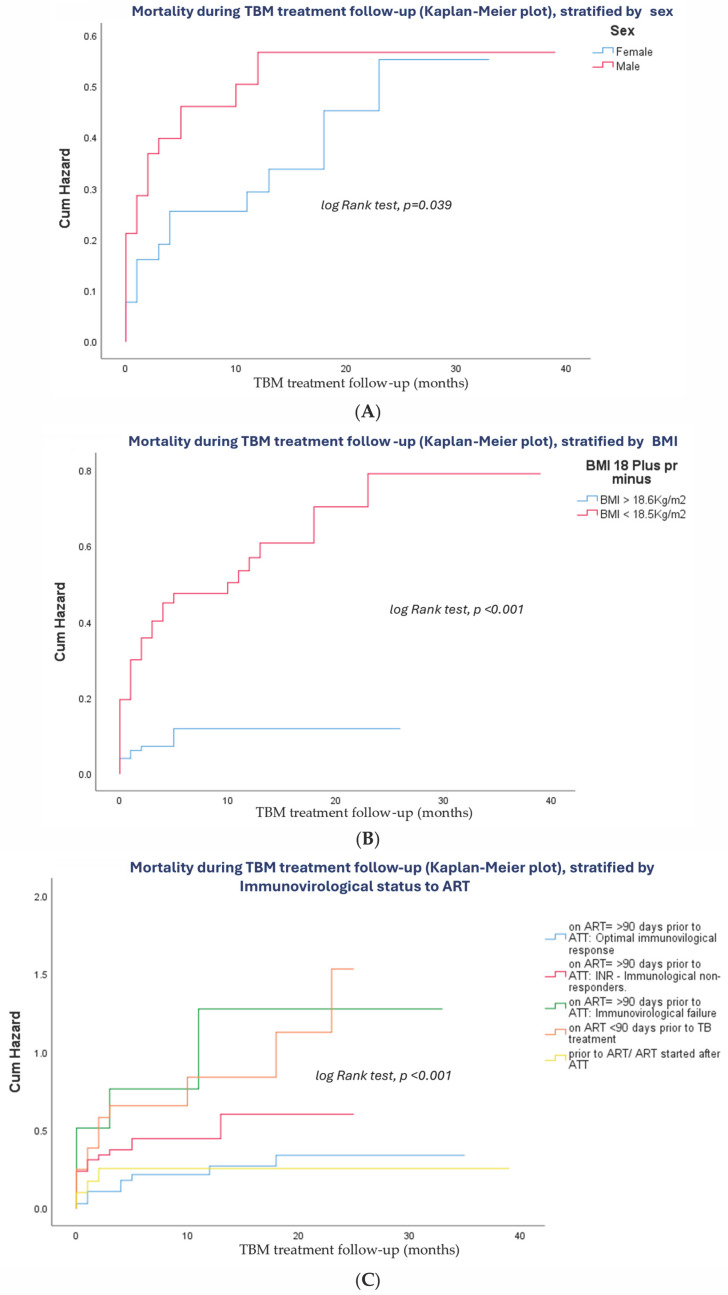

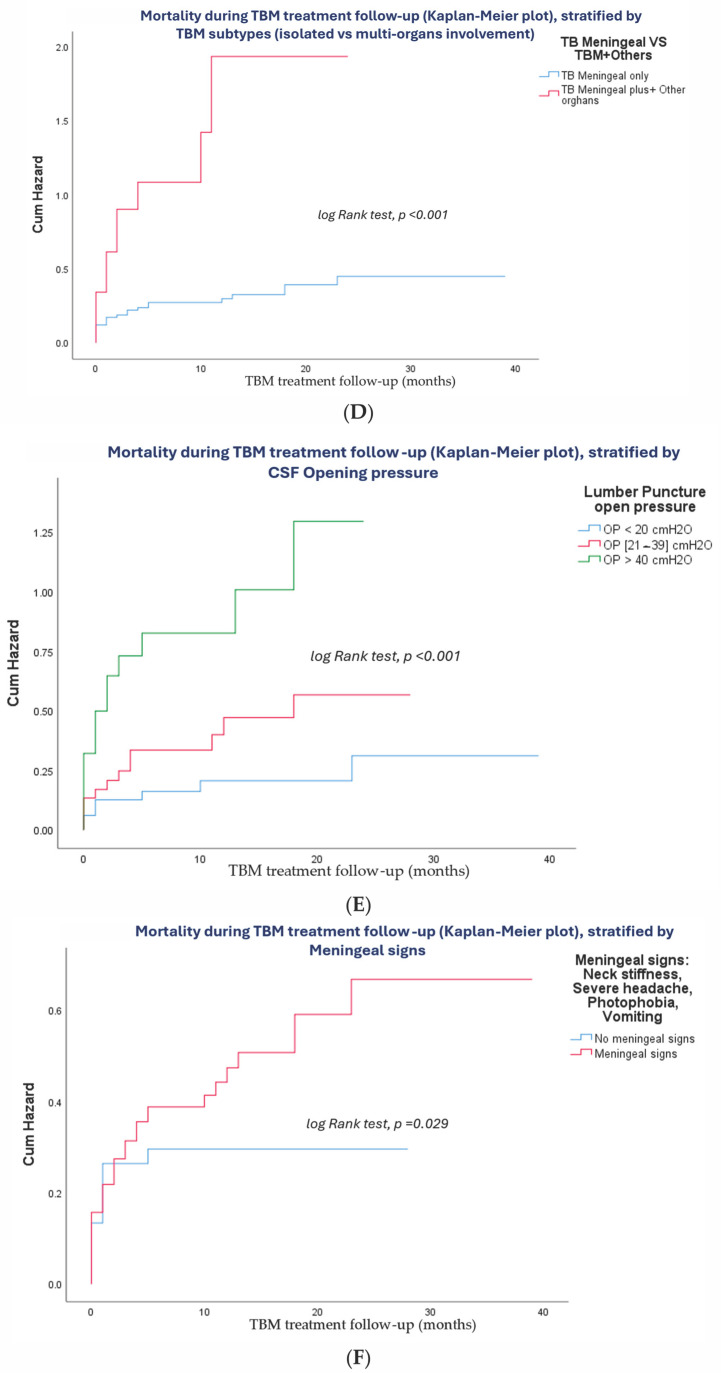

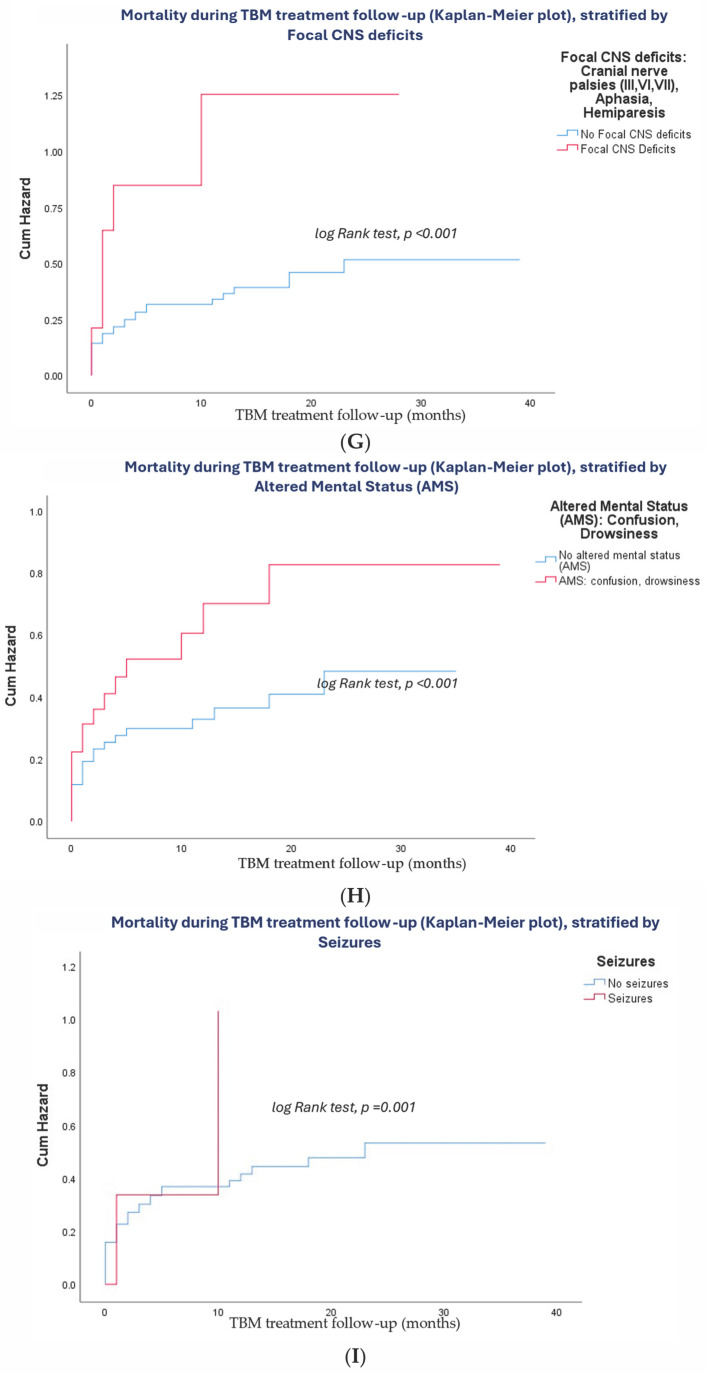

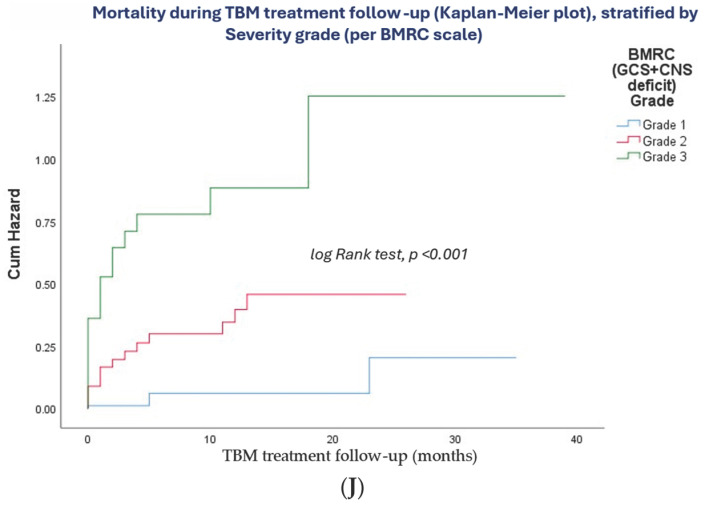
(**A**) PTBM men had a higher cumulative incidence of death above 40% after three months of follow-up (log-rank test, *p* = 0.039). (**B**) Persons with TBM TB with a body mass index below 18.5 g/dL had a higher cumulative incidence of death above 40% after three months of follow-up (log-rank test, *p* = <0.001). (**C**) Those on 90 days of ART prior to ATT with immunological failure had a higher cumulative incidence of death above 50% after three months of follow-up (log-rank test, *p* = <0.001). (**D**) Those presenting with TBM with other organs involved had a higher cumulative incidence of death above 90% after three months of follow-up (log-rank test, *p* = <0.001). (**E**) Those presenting open pressure lumbar puncture more or equal to 40 cmH20 had a higher cumulative incidence of death above 65% after three months of follow-up (log-rank test *p* = <0.001). (**F**) Those presenting meningeal signs had a higher cumulative incidence of death above 31.5% after three months of follow-up (log-rank test *p* = 0.029). (**G**) Those presenting focal CNS deficits had a higher cumulative incidence of death above 85% after three months of follow-up (log-rank *p* = <0.001). (**H**) Those presenting altered mental status had a higher cumulative incidence of death above 36% after three months of follow-up (log-ank *p* = <0.001). (**I**) Those presenting with seizures had a higher cumulative incidence of death above 34% after three months of follow-up (log-rank test *p* = 0.001). (**J**) Those presenting TBM severity grade III (per the BMRC scale) had a higher cumulative incidence of death above 64.5% after three months of follow-up (log-rank test *p* = <0.001).

A Kaplan–Meier survival analysis was performed to evaluate time-to-death among non-survivors (*n* = 140); Figure 3 presents the Kaplan–Meier cumulative probability of mortality among PTBM at CHC stratified by key predictors:

PTBM men had a higher cumulative incidence of death than women, exceeding 40% by three months of follow-up (*p* = 0.039, Figure 3A). Undernourished PTBM (BMI < 18.5 kg/m^2^) experienced significantly greater mortality, with cumulative deaths surpassing 40% within three months of follow-up (*p* < 0.001, Figure 3B). PTBM with immunovirological failure despite being on ART for more than 90 days prior to TB treatment initiation had over 50% mortality by three months of follow-up (*p* < 0.001, Figure 3C). Those who had TBM with concurrent involvement of other organs had the highest cumulative mortality, exceeding 90% at three months of follow-up (*p* < 0.001, Figure 3D).

Elevated CSF OP was another strong predictor of early death; PTBM with OP ≥ 40 cmH_2_O had cumulative mortality above 65% (*p* < 0.001, Figure 3E). The presence of meningeal signs was also associated with worse survival, with a three-month mortality rate of over 31.5% compared to those without such signs (*p* = 0.029, Figure 3F). Focal neurological deficits, including cranial nerve palsies and hemiparesis, significantly increased the risk of death, with cumulative incidence exceeding 85% within three months of follow-up (*p* < 0.001, Figure 3G). Similarly, altered mental status was linked to a higher risk of early mortality, with over 36% dying by three months of follow-up (*p* < 0.001, Figure 3H).

Seizures, although less common, were associated with a three-month cumulative mortality above 34% (*p* = 0.001, Figure 3I). Finally, PTBM with TBM severity grade III (BMRC scale) had markedly worse outcomes, with more than 64.5% dying within the first three months of follow-up (*p* < 0.001, Figure 3J).

## 4. Discussion

This study presents a comprehensive analysis of mortality and its associated predictors among PTBM at a rural hospital in Mozambique over a six-year period, providing context-specific insights that could inform future clinical strategies and programmatic responses for managing TBM.

The overall mortality rate of 37.6% highlights the severe prognosis of TBM in resource-limited settings and is consistent with findings from other high-burden TB/HIV countries, where diagnostic delays, limited access to neuroimaging, and advanced disease presentation remain common challenges [30,31].

### 4.1. PTBM Men

The original study data revealed a significant sex disparity in mortality outcomes among PTBM, with men accounting for 60% of deaths (84 of 140) compared to 40% among women (56 of 140). This finding was further supported by the aHR of 1.80 (1.21–2.68) for men, underscoring their elevated risk of mortality. While broader meta-analyses report an overall PTBM case fatality rate of 38.1%, sex-stratified data remain limited [32]. However, corroborating evidence from Kenya showed higher mortality in PTBM men (11%) versus women (9%), aligning with the observed trend [32]. Although studies such as the Romanian cohort did not explicitly analyze sex differences, the collective evidence suggests that PTBM men face worse outcomes, likely influenced by biological, behavioral, or healthcare-related factors [33]. The consistent pattern of higher mortality among PTBM men across multiple studies points to potential underlying drivers. While global data gaps exist, regional findings—such as the Kenyan study, demonstrating a lower aHR of 0.83 for women—reinforce the sex-based survival difference [34]. This disparity may stem from a combination of factors, including later healthcare-seeking behavior in men, a higher prevalence of comorbid conditions (e.g., substance use), or biological differences in disease progression [34]. The lack of sex-disaggregated data in some studies highlights the need for further research to clarify these mechanisms and inform targeted interventions for at-risk populations.

### 4.2. PTBM with Undernutrition

The findings from our study, which revealed a 92.1% mortality rate among PTBM with undernutrition (BMI < 18.5 kg/m^2^) and an aHR of 2.75, align with broader research highlighting malnutrition as a critical predictor for TBM outcomes. Globally, studies in Asia and Latin America have similarly identified low BMI as a predictor of higher mortality in TBM, with meta-analyses reporting pooled aHRs of 2.1–2.3 for undernutrition in HIV/TB co-infected populations [35,36]. In sSA, where malnutrition and HIV/TB co-infections are endemic, the interplay between undernutrition and TBM mortality is even more pronounced. For instance, a South African cohort reported a 67% mortality rate among PTBM with severe malnutrition (BMI < 16 kg/m^2^), while a Mozambican study linked undernutrition to delayed ART initiation and poorer immunological recovery, exacerbating TBM severity [37,38,39]. Our results thus reinforce the global consensus that undernutrition significantly worsens TBM prognosis, particularly in high-burden, resource-limited settings.

The stark association between undernutrition and TBM mortality underscores the urgent need for integrated nutritional interventions in TBM management protocols. Clinically, routine BMI screening and therapeutic feeding programs should be prioritized for PTBM, as evidenced by studies showing reduced mortality with macronutrient supplementation [36,40]. Research must further explore the biological mechanisms linking malnutrition to blood–brain barrier permeability and immune dysfunction in TBM, as well as the efficacy of targeted interventions such as high-energy diets or micronutrient fortification [37,41]. At the policy level, our findings advocate for national TB programs in SSA to adopt “double-duty” actions—such as combining nutritional support with ART and TB therapy—to address the syndemic of HIV, TB, and food insecurity [39,42]. Strengthening food security initiatives, particularly for high-risk groups including farmers and young adults (identified as TBM-vulnerable in Chinese and African cohorts [43]), could mitigate the dual burden of malnutrition and infectious disease. These measures align with WHO goals to reduce TB mortality by 2030, emphasizing the need for multisectoral collaboration to break the cycle of poverty, malnutrition, and TB in endemic regions.

### 4.3. PTBM with Immunovirological Failure to ART

The findings from our study in Mozambique align with and expand upon existing global and SSA research on TBM. The mortality incidence among PTBM with immunovirological failure to ART (22.22 per 100 person-months; aHR = 2.82) was significantly higher than that reported in other regions, such as Southeast Asia, where TBM mortality in HIV-coinfected patients ranged from 27% to 51% [44,45]. In SSA, pooled short-term mortality for TBM in HIV-positive individuals was 46% in routine care settings, reflecting similar challenges in delayed diagnosis and limited treatment access [11,32]. Our study also corroborates findings from South Africa, where advanced immunosuppression (CD4 < 100 cells/µL) and malnutrition were key predictors of poor outcomes [46,47]. However, the mortality incidence in our cohort exceeded that of high-burden countries such as Vietnam, where optimized ART adherence reduced mortality to 23% in HIV-uninfected patients [45]. These disparities highlight the compounded impact of HIV-related immunosuppression, undernutrition, and healthcare access disparities in rural Mozambique compared to urban centers with better resources [32,47].

The high mortality linked to immunovirological failure underscores the urgent need for integrated HIV/TB care strategies in resource-limited settings. Clinically, early ART initiation with viral load monitoring and nutritional support should be prioritized for PTBM, particularly those with BMI < 18.5 kg/m^2^ [32,47]. Research must focus on rapid diagnostics (e.g., Xpert Ultra) and host-directed therapies to mitigate CNS inflammation, as current regimens fail to address the high mortality in advanced HIV [48,49]. Policy-wise, our findings advocate for: (1) decentralized TBM diagnostic tools in rural hospitals, (2) task-shifting to empower frontline healthcare workers in managing intracranial hypertension, and (3) the inclusion of TBM-specific indicators in national TB/HIV programs to improve surveillance [50,51]. Addressing these gaps could align Mozambique’s response with WHO targets to reduce TB mortality by 75% by 2025 [44,49].

### 4.4. TBM with Multi-Organ Involvement

The findings from our study, which reported a mortality rate of 33.6% (aHR = 2.07) for TBM with multi-organ involvement and 90% cumulative mortality at three months, align with but also contrast trends observed in other regions. In SSA, a meta-analysis revealed an overall TBM mortality rate of 25%, escalating to 70% in high-HIV-burden areas such as Africa, underscoring the impact of comorbidities and delayed diagnosis [52]. Similarly, a retrospective cohort in Iran documented an 18% in-hospital mortality rate, with hydrocephalus (HR = 3.65) and altered consciousness (HR = 19.23) as key predictors, though multi-organ involvement was not explicitly analyzed [30]. In contrast, studies from Asia, such as one in Shenzhen, China, emphasized that microbiologically confirmed TBM cases had worse outcomes linked to malnutrition and HIV co-infection, though mortality rates were lower than in our cohort [53]. These disparities highlight regional variations in healthcare access, diagnostic capacity, and comorbid conditions influencing TBM outcomes.

The high mortality associated with multi-organ TBM in our study underscores the urgency of early diagnosis and aggressive management, particularly in resource-limited settings. Clinically, the findings advocate for integrating advanced diagnostics (e.g., Xpert Ultra, mNGS, and point-of-care ultrasound techniques [54]) to detect multi-organ involvement promptly, as delays exacerbate mortality risks [1,55,56]. Research should prioritize validating biomarkers (e.g., CSF APOB, NELL2) and shorter, more effective regimens (e.g., BPaL for drug-resistant TBM) to improve survival [1,55,56]. Policy-wise, the data call for strengthened TB surveillance systems in high-burden regions, alongside targeted interventions for immunocompromised populations, such as optimized ART adherence in HIV co-infected patients [50,52]. Public health strategies must also address malnutrition and healthcare access disparities, which amplify TBM severity, as evidenced by the stark mortality differences between our cohort and better-resourced settings [52,53].

### 4.5. TBM with Intracranial Pressure (ICP) OP > 40 cmH_2_O

The findings from our study align with global and SSA research on TBM, which consistently highlights elevated ICP as a critical predictor of mortality. In our cohort, patients with ICP > 40 cmH_2_O had a mortality rate of 45.7% (aHR = 3.83), mirroring trends observed in other high-burden regions. A meta-analysis of TBM in SSA reported a pooled short-term mortality of 46% (95% CI: 33–59%) in routine care settings, underscoring the lethal impact of delayed diagnosis and inadequate ICP management [11]. Similarly, global studies note that TBM-associated hydrocephalus and vasculitis—common in high-ICP cases—contribute to mortality rates exceeding 40% in hospitalized patients [57]. Our results reinforce the consensus that severe ICP in TBM is a near-universal marker of poor prognosis, particularly in resource-limited settings where advanced neurocritical care is scarce.

The high mortality linked to ICP > 40 cmH_2_O underscores the urgent need for improved TBM management protocols, particularly in endemic regions. Clinically, early ICP monitoring and interventions—such as therapeutic lumbar punctures, hyperosmolar agents (e.g., mannitol), and corticosteroids—should be prioritized to mitigate secondary brain injury [58,59]. Research must focus on validating scalable ICP-lowering strategies, including the use of hypertonic saline in low-resource settings, as suggested by experimental models. Policy-wise, integrating TBM-specific guidelines into national TB programs is critical, as is emphasizing rapid diagnostic algorithms (e.g., Xpert Ultra for CSF) and adjunctive therapies. Additionally, our findings advocate for enhanced surveillance of TBM outcomes in high-HIV-burden areas, where immunosuppression exacerbates ICP-related mortality [44,57]. Addressing these gaps could significantly advance progress toward the WHO’s End TB targets by reducing preventable TBM deaths.

### 4.6. TBM with Meningeal Signs

The findings from our study, where meningeal signs were associated with 82.9% of deaths (aHR = 2.87), align with global trends but reveal higher mortality rates compared to some regions. In SSA, TBM mortality often exceeded 50%, particularly in HIV-endemic areas, whereas studies from Asia and Europe reported lower rates (20–40%) [60,61,62]. Our results mirrored the high fatality rates observed in Ethiopia (33.9% mortality in children) and Mozambique (37.6% overall), underscoring the impact of delayed diagnosis and advanced disease stages in resource-limited settings [60,61]. Notably, the strong association between meningeal signs and death in our cohort was consistent with reports from South Africa and China, where neurological complications (e.g., hydrocephalus, vasculitis) similarly worsened outcomes [61]. However, our adjusted hazard ratio (aHR = 2.87) was higher than in some Asian cohorts (aHR of 1.5–2.0), possibly due to differences in healthcare access or comorbid HIV prevalence [62].

The high mortality linked to meningeal signs underscores the need for early intervention protocols in clinical practice, particularly in regions with limited diagnostic tools. Prioritizing patients with meningeal symptoms for aggressive management (e.g., corticosteroids, ICP monitoring) could mitigate poor outcomes, as suggested by studies from China and Ethiopian [61,62]. In terms of research, our findings highlight gaps in biomarker development for rapid TBM diagnosis, given the limitations of CSF culture sensitivity (29.7% pooled yield) [62]. Investment in point-of-care tools (e.g., enhanced GeneXpert Ultra, portable ultrasound) is critical, especially in SSA, where diagnostic delays are prevalent [50,54,62]. Policy-wise, integrating TBM screening into national TB/HIV programs—akin to Ethiopia’s “End TB Strategy”—could improve early detection [50,60]. Additionally, malnutrition (aHR = 2.75 in our study) and HIV co-infection demand targeted public health interventions, such as nutritional support and optimized ART regimens, to address modifiable predictors [60,63].

### 4.7. TBM with Focal CNS Deficits

Our study found that focal CNS deficits were present in 20.0% of deaths, with a cHR of 2.80, though this association lost significance after adjustment. Additionally, patients with focal CNS deficits had an 85% cumulative mortality rate at three months, highlighting the severe prognosis associated with neurological complications. These findings align with global trends, where TBM-related mortality remains high, particularly in low-resource settings. For instance, a meta-analysis of TBM mortality reported an average case fatality rate of 27% in treated patients, with neurological sequelae contributing significantly to poor outcomes [62]. In SSA, where HIV coinfection and delayed diagnosis are prevalent, studies report even higher mortality rates (up to 50%) due to advanced disease at presentation and limited access to neuroimaging for the early detection of complications such as infarcts and hydrocephalus [64,65]. Compared to high-income countries, where mortality ranges between 15 and 30%, our findings underscore the disproportionate burden of TBM in regions with constrained healthcare infrastructure [63].

The high mortality linked to focal CNS deficits in our study calls for urgent clinical and public health interventions. Early neuroimaging (MRI/CT) should be prioritized in high-risk populations to detect infarcts, tuberculomas, or hydrocephalus, enabling timely neurosurgical or medical management [50,65]. Additionally, enhanced diagnostic tools, such as Xpert MTB/RIF Ultra (sensitivity: 44% in CSF), could improve early detection, particularly in HIV-endemic regions where paucibacillary CSF complicates diagnosis [62]. From a research perspective, prospective studies are needed to validate whether aggressive anti-inflammatory therapies (e.g., corticosteroids, TNF-α inhibitors) or intrathecal drug delivery (e.g., nanoparticle-infused hydrogels) can mitigate neurovascular complications [65,66]. Policy-wise, integrating TBM screening into TB/HIV programs and expanding access to BCG vaccination—particularly in high-incidence areas—could reduce incidence and severity [50]. Finally, global guidelines should emphasize risk stratification models incorporating focal neurological signs to guide resource allocation in endemic regions [62,64].

### 4.8. TBM with Altered Mental Status (AMS)

The findings from our study, where AMS was associated with 42.9% of deaths (cHR = 1.78, though not significant after adjustment), align with and contrast against global and SSA research. In a study from China, AMS was a significant predictor of mortality in stage II/III TBM, with advanced age and low GCS scores further exacerbating outcomes [41]. Similarly, research in South Africa and Uganda highlighted that AMS, alongside HIV co-infection and high ICP, contributed to mortality rates exceeding 50% in high-burden settings [44,67]. However, our adjusted analysis diverged from these studies, where AMS remained independently significant (e.g., aHR = 2.87 for meningeal signs in Mozambique [47]). Discrepancies may stem from differences in healthcare access, diagnostic delays, or ART adherence, as SSA studies often report higher mortality due to late-stage presentation and resource limitations [65].

The persistent association of AMS with mortality underscores the need for early neurological monitoring in TBM management, particularly in high-burden regions. Clinically, integrating AMS assessment with other high-predictors (e.g., low BMI, high ICP) could refine prognostic models and guide aggressive interventions, such as intensified anti-TB regimens or corticosteroids, as trialed in the INTENSE-TBM project [67]. For research, our findings highlight gaps in understanding why AMS loses significance after adjustment—possibly due to confounding variables such as HIV status or drug resistance—warranting larger, multicenter studies [68]. Policy-wise, the data advocate for strengthened TBM surveillance in SSA, improved ART integration, and capacity-building for rapid diagnostics, as delayed treatment remains a critical driver of poor outcomes [44,67]. Addressing malnutrition (BMI <18.5) and optimizing HIV care could further mitigate mortality, aligning with WHO goals to reduce TB-related deaths by 2030 [65,69].

### 4.9. TBM with Severity Grade III (per BMRC Scale)

The findings from our study align with global and SSA data, which consistently highlight the high mortality associated with severe TBM. For instance, a meta-analysis of TBM cases reported a 42.1% mortality rate in hospitalized patients, with SSA experiencing rates as high as 70% among PLWH with TB [57,70]. Our observation that 55.7% of deaths occurred in patients with BMRC grade III TBM and low GCS scores (aHR = 2.15) mirrors trends from South Africa and Uganda, where severe neurological deficits and hydrocephalus were key predictors of poor outcomes [41,71]. Similarly, a global modeling study estimated a 48% mortality rate for incident TBM, with the highest burden in Southeast Asia and Africa, reinforcing the regional disparities in outcomes [44]. The 64.5% cumulative mortality rate at three months in our cohort parallels findings from the INTENSE-TBM trial, where late-stage TBM mortality exceeded 50% despite standard treatment [70,71].

The high mortality linked to advanced TBM underscores the urgent need for early diagnosis and intensified treatment regimens. Our results support the adoption of high-dose rifampicin (35 mg/kg) and adjunctive linezolid, as trialed in the INTENSE-TBM study, to improve CSF drug penetration and reduce mortality by 30% [70,71]. Policy efforts should prioritize integrating TBM screening into national TB programs, particularly in high-HIV settings, and expanding access to rapid diagnostics such as Xpert Ultra, which has 44% sensitivity for TBM [49,62]. Research should focus on host-directed therapies (e.g., aspirin for vasculitis) and optimized ART timing in HIV-coinfected patients, given the 25.7% mortality observed in those initiating ART < 90 days before TB treatment [50,68]. Finally, neuroprotective strategies for patients with hydrocephalus or low GCS scores could mitigate disability, as 50% of survivors suffer long-term sequelae [44,71].

### 4.10. Strength and Limitations

This study has several strengths. It included a relatively large number of PTBM over a six-year period, which allowed for meaningful analysis of mortality patterns in a rural hospital setting. By using routinely collected clinical data, the study reflects real-world conditions in a resource-limited environment. A key strength was the ability to differentiate between subgroups of HIV co-infected patients, such as those with optimal immune responses, immunological non-responses, and immunovirological failure—an area not often explored in detail. The study also incorporated a broad range of clinical indicators, including CSF opening pressure, neurological status, ART history, BMI, and the extent of TB involvement, to better understand factors associated with poor outcomes. The use of survival analysis and multivariable Cox regression helped to identify independent predictors of mortality while accounting for potential confounders. Additionally, the study contributes to the limited evidence on TBM outcomes among patients with disseminated TB, highlighting an area that may require further research.

There are, however, several limitations. First, the study’s retrospective design, based on routine clinical records, made it susceptible to missing or incomplete data, which may have introduced misclassification or limited our ability to fully adjust for confounders. Second, this study is limited by selection bias, as the use of a laboratory logbook for case identification means we only included patients who underwent CSF culture, thus excluding any patients with CNS symptoms who were not tested and precluding an evaluation of TBM prevalence or clinical screening practices. Third, the single-center design may limit generalizability to other healthcare settings in Mozambique or the broader sub-Saharan African context.

Lastly, the study was unable to evaluate the average (mean/median) hospitalization duration for survivors due to data limitations. Although the enrollment date corresponded with hospital admission, the date of cure did not consistently match the discharge date. Many patient records lacked complete hospitalization timeline data, making it impossible to calculate the length of ward stay

For this specific study, we did not include comorbidities (such as chronic inflammatory/metabolic conditions such as diabetes and hypertension, or neoplasms) as a variable, but we plan to conduct separate studies focusing on their impact on TB outcomes.

## 5. Conclusions and Recommendations

Based on the comprehensive analysis of mortality predictors among persons with HIV and TBM in rural Mozambique, the study concluded that immunovirological failure to ART and severe ICP (CSF OP > 40 cmH_2_O) were the most critical predictors, necessitating urgent interventions such as ART optimization and emergent CSF pressure management. Malnutrition (BMI < 18.5 kg/m^2^) was a high-priority systemic risk, requiring aggressive nutritional support, while neurological complications (TBM severity grade III, focal deficits) warranted corticosteroids and seizure prophylaxis to mitigate irreversible damage. Men were at heightened risk, likely due to delays in care-seeking, and they warranted enhanced monitoring. The study affirms that an integrated clinical approach should have prioritized acute, life-threatening factors (immunological and ICP crises), followed by addressing malnutrition, and finally managing neurological sequelae, to ensure a balance between immediate survival and long-term recovery. These findings underscore the need for early diagnosis, prompt LP with pressure assessment, and integrated HIV, TB, and nutritional support services in resource-limited settings to reduce the high mortality burden of TBM.

## Figures and Tables

**Table 1:** Demographic and clinical characteristics of 372 persons with tuberculosis meningitis at the TB Clinic (CHC) in Mozambique, 2015–2020, according to mortality outcome.

	Total N (%)	Death N (%)	Person-Months (PM)	Incidence per PM (95% CI)
Total	372 (100.0)	140 (37.63)	3720	3.76 (3.14–3.76)
**Treatment follow-up**				
Treatment follow-up (median, [IQR])	10 [2; 20]			
**Sex**				
Women	162 (43.5)	56 (40.0)	2430	2.30 (1.92–2.66)
Men	210 (56.5)	84 (60.0)	1890	4.44 (4.00–4.44)
**Age**				
Age (median, [IQR])	36 [29; 42]			
**Standard Age Group for TB Reporting**				
0–14	10 (2.7)	0 (0.0)	200	0.0 (0.0–0.0)
15–24	42 (11.3)	12 (8.6)	756	1.59 (1.36–2.38)
25–34	104 (28.0)	44 (31.4)	988	4.45 (3.53–5.29)
35–44	140 (37.6)	52 (37.1)	1400	3.71 (3.10–4.13)
45–54	42 (11.3)	16 (11.4)	420	3.81 (1.90–5.08)
55–64	26 (7.0)	12 (8.6)	182	6.59 (4.62–9.23)
65+	8 (2.2)	4 (2.9)	36	11.11 (4.55–21.84)
**BMI (Body Mass Index)**				
BMI > 18.6 kg/m^2^	101 (27.2)	11 (7.9)	1111	0.99 (0.64–1.09)
BMI ≤ 18.5 kg/m^2^	271 (72.8)	129 (92.1)	2710	4.76 (3.97–5.29)
**Immunovirological Status to ART Prior to ATT**				
on ART ≥ 90 days prior to ATT: Optimal immunovirological response	139 (37.4)	34 (24.3)	1668	2.04 (1.29–2.22)
on ART ≥ 90 days prior to ATT: INR—Immunological non-responders.	90 (24.2)	36 (25.7)	810	4.44 (3.33–5.00)
on ART ≥ 90 days prior to ATT: Immunovirological failure	30 (8.1)	20 (14.3)	90	22.22 (6.06–22.22)
on ART < 90 days prior to TB treatment	50 (13.4)	36 (25.7)	450	8.00 (4.80–36.00)
prior to ART/ART started after ATT	63 (16.9)	14 (10.0)	693	2.02 (1.39–2.22)
**TB Meningeal vs. TBM + Others**				
TB meningeal only	313 (84.1)	93 (66.4)	3756	2.48 (1.98–2.70)
TB meningeal plus+ other organs	59 (15.9)	47 (33.6)	118	39.83 (8.85–39.83)
**CSF Open Pressure Lumbar Puncture**				
OP ≤ 20 cmH_2_O	136 (37.2)	28 (20.0)	1768	1.58 (1.08–1.87)
OP [21,22,23,24,25,26,27,28,29,30,31,32,33,34,35,36,37,38,39] cmH_2_O	128 (35.0)	48 (34.3)	1344	3.57 (3.13–4.17)
OP ≥ 40 cmH_2_O	102 (27.9)	64 (45.7)	204	31.37 (12.55–62.75)
**Meningeal signs: (Neck stiffness, Severe headache, Photophobia, Vomiting)**				
No meningeal signs	96 (25.8)	24 (17.1)	864	2.78 (2.08–2.78)
Meningeal signs	276 (74.2)	116 (82.9)	3036	3.82 (3.23–4.20)
**Focal CNS deficits: (Cranial nerve palsies [III, VI, VII], Aphasia, Hemiparesis)**				
No Focal CNS deficits	330 (88.7)	112 (80.0)	3630	3.09 (2.61–3.39)
Focal CNS Deficits	42 (11.3)	28 (20.0)	84	33.33 (7.41–66.67)
**Altered Mental Status (AMS): Confusion, Drowsiness**				
No altered mental status (AMS)	252 (67.7)	80 (57.1)	2772	2.89 (2.44–2.89)
AMS: confusion, drowsiness	120 (32.3)	60 (42.9)	1080	5.56 (4.17–5.56)
**Seizures**				
No seizures	356 (96.2)	128 (91.4)	3916	3.27 (2.77–3.60)
Seizures	14 (3.8)	12 (8.6)	140	8.57 (4.76–8.57)
**Modified British Medical Research Council (BMRC) Scale**				
Grade I (GCS 15, no focal deficits)	86 (23.1)	9 (6.4)	1634	0.55 (0.48–0.58)
Grade II (GCS 11–14 or 15 with focal deficits)	164 (44.1)	53 (37.9)	1804	2.94 (2.49–3.23)
Grade III (GCS ≤ 10)	122 (32.8)	78 (55.7)	366	21.31 (7.10–31.97)

IQR: interquartile range, GCS: Glasgow Coma Scale, CSF: Cerebrospinal fluid, CNS: central nervous system, ATT: anti-TB treatment.

**Table 2:** Cox proportional hazards model for mortality in 372 persons with Tuberculosis meningitis at the TB clinic (CHC) in Mozambique, 2015–2020.

	Crude cHR (95% CI)	*p*-Value	Adjusted aHR (95% CI)	*p*-Value
**Sex**				
Women	Reference		Reference	
Men	1.41 (1.00–1.98)	0.048	1.80 (1.21–2.68)	0.004
**Standard Age Group for TB Reporting**				
25–34	Reference			
0–14	0.00 (0.00–0.00)	0.950		
15–24	0.55 (0.29–1.04)	0.067		
35–44	0.90 (0.60–1.34)	0.589		
45–54	0.98 (0.55–1.74)	0.949		
55–64	1.26 (0.66–2.39)	0.478		
65+	1.78 (0.64–4.96)	0.273		
**BMI (Body Mass Index)**				
BMI > 18.6 kg/m^2^	Reference		Reference	
BMI ≤ 18.5 kg/m^2^	5.03 (2.72–9.32)	<0.001	2.84 (1.46–5.55)	0.002
**Immunovirological Status to ART Prior to ATT**				
on ART = >90 days prior to ATT: Optimal immunovirological response	Reference		Reference	
on ART = >90 days prior to ATT: INR—Immunological non-responders.	1.99 (1.25–3.19)	0.004	2.06 (1.23–3.46)	0.006
on ART = >90 days prior to ATT: Immunovirological failure	3.88 (2.23–6.76)	<0.001	2.86 (1.56–5.23)	0.001
on ART < 90 days prior to TB treatment	3.55 (2.22–5.68)	<0.001	2.09 (1.24–3.52)	0.006
prior to ART/ART started after ATT	0.96 (0.51–1.78)	0.886	0.69 (0.36–1.32)	0.262
**TB Meningeal vs. TBM + Others**				
TB Meningeal only	Reference		Reference	
TB Meningeal plus+ Other organs	4.27 (2.97–6.15)	<0.001	2.03 (1.27–3.25)	0.003
**CSF Open Pressure (OP) Lumbar Puncture**				
OP ≤ 20 cmH_2_O	Reference		Reference	
OP [21,22,23,24,25,26,27,28,29,30,31,32,33,34,35,36,37,38,39] cmH_2_O	2.01 (1.26–3.20)	0.003	1.40 (0.75–2.64)	0.295
OP ≥ 40 cmH_2_O	4.47 (2.85–7.01)	<0.001	2.67 (1.46–4.86)	0.001
**Meningeal signs: (Neck stiffness, Severe headache, Photophobia, Vomiting)**				
No meningeal signs	Reference		Reference	
Meningeal signs	1.59 (1.03–2.47)	0.038	2.66 (1.60–4.41)	<0.001
**Focal CNS deficits: (Cranial nerve palsies [III, VI, VII], Aphasia, Hemiparesis)**				
No Focal CNS deficits	Reference		Reference	
Focal CNS Deficits	2.80 (1.83–4.26)	<0.001	1.33 (0.74–2.39)	0.336
**Altered Mental Status (AMS): (Confusion, Drowsiness)**				
No altered mental status (AMS)	Reference		Reference	
AMS: confusion, drowsiness	1.78 (1.27–2.48)	0.001	1.06 (0.69–1.62)	0.793
**Seizures**				
No seizures	Reference		Reference	
Seizures	2.50 (1.38–4.53)	0.002	0.99 (0.66–1.52)	0.994
**Modified British Medical Research Council (BMRC) Scale**				
Grade I (GCS 15, no focal deficits)	Reference		Reference	
Grade II (GCS 11–14 or 15 with focal deficits)	3.80 (1.87–7.71)	<0.001	2.35 (0.98–5.78)	0.065
Grade III (GCS ≤ 10)	9.61 (4.81–19.21)	<0.001	4.59 (1.79–11.76)	0.001

GCS: Glasgow Coma Scale, CSF: Cerebrospinal fluid, CNS: central nervous system, ATT: anti-TB treatment, aHR: adjusted hazard ratio, cHR: crude hazard ratio.

## Data Availability

The datasets utilized in this study are available from the corresponding author upon reasonable request; however, they are not publicly accessible due to privacy constraints.

## Abbreviations

The following abbreviations are used in this manuscript:

aHR	Adjusted hazard ratios
ART	Antiretroviral therapy
ATT	Anti-TB Treatment
BMI	Body mass index
BMRC	British Medical Research Council
CI	Confidence intervals
CSF	Cerebrospinal fluid
CHC	Carmelo Hospital of Chókwè
CXR	Chest X-ray examination
GCS	Glasgow Coma Scale
IQR	Interquartile range
INR	Immunological non-responders
IVF	Immunovirological failure
OIVR	Optimal immunovirological response
OP	Opening pressure
PLWH	Person living with HIV
PTBM	PLWH with Tuberculous meningitis
LP	Lumbar puncture
NAAT	Nucleic Acid Amplification Test
sSA	Sub-Saharan Africa
TBM	Tuberculous meningitis
WHO	World Health Organization
